# Genome-Wide Association of Implantable Cardioverter-Defibrillator Activation With Life-Threatening Arrhythmias

**DOI:** 10.1371/journal.pone.0025387

**Published:** 2012-01-11

**Authors:** Sarah S. Murray, Erin N. Smith, Nikki Villarasa, Tara Nahey, Jeff Lande, Harold Goldberg, Marian Shaw, Lawrence Rosenthal, Brian Ramza, Jamshid Alaeddini, Xinqiang Han, Samir Damani, Orhan Soykan, Robert C. Kowal, Eric J. Topol, on behalf of the GAME Investigators

**Affiliations:** 1 Scripps Genomic Medicine, Scripps Translational Science Institute, La Jolla, California, United States of America; 2 Baylor Heart and Vascular Hospital, Baylor University Medical Center, Dallas, Texas, United States of America; 3 Ventures and New Therapies, Medtronic, Inc, Fridley, Minnesota, United States of America; 4 University of Massachusetts, Worcester, Massachusetts, United States of America; 5 St. Luke's Health System, Mid America Heart Institute, Kansas City, Missouri, United States of America; 6 Inland Cardiology Associates, Spokane, Washington, United States of America; 7 Richmond Cardiology Associates, Richmond, Indiana, United States of America; 8 Department of Pediatrics and Rady's Children's Hospital, University of California at San Diego, School of Medicine, La Jolla, California, United States of America; 9 Sequenom, Inc., San Diego, California, United States of America; Emory University School Of Medicine, United States of America

## Abstract

**Objectives:**

To identify genetic factors that would be predictive of individuals who require an implantable cardioverter-defibrillator (ICD), we conducted a genome-wide association study among individuals with an ICD who experienced a life-threatening arrhythmia (LTA; cases) vs. those who did not over at least a 3-year period (controls).

**Background:**

Most individuals that receive implantable cardioverter-defibrillators never experience a life-threatening arrhythmia. Genetic factors may help identify who is most at risk.

**Methods:**

Patients with an ICD and extended follow-up were recruited from 34 clinical sites with the goal of oversampling those who had experienced LTA, with a cumulative 607 cases and 297 controls included in the analysis. A total of 1,006 Caucasian patients were enrolled during a time period of 13 months. Arrhythmia status of 904 patients could be confirmed and their genomic data were included in the analysis. In this cohort, there were 704 males, 200 females, and the average age was 73.3 years. We genotyped DNA samples using the Illumina Human660 W Genotyping BeadChip and tested for association between genotype at common variants and the phenotype of having an LTA.

**Results and Conclusions:**

We did not find any associations reaching genome-wide significance, with the strongest association at chromosome 13, rs11856574 at P = 5×10^−6^. Loci previously implicated in phenotypes such as QT interval (measure of the time between the start of the Q wave and the end of the T wave as measured by electrocardiogram) were not found to be significantly associated with having an LTA. Although powered to detect such associations, we did not find common genetic variants of large effect associated with having a LTA in those of European descent. This indicates that common gene variants cannot be used at this time to guide ICD risk-stratification.

**Trial Registration:**

ClinicalTrials.gov NCT00664807

## Introduction

Sudden cardiac arrest death (SCD) accounts for the loss of over 300,000 individuals each year in the United States [1] and approximately 80% of those affected have underlying coronary artery disease [1]. Many studies have provided evidence that there is a genetic contribution to SCD by demonstrating family history as being a risk factor for sudden cardiac death or cardiac arrest [2], [3], [4]. In addition, multiple family studies have emphasized the importance of heritability in SCD, with relative risks of 1.5 to 2.7 in case-control studies among first-degree relatives of individuals who have died suddenly [5], [6].

Recently, several studies have demonstrated specific gene variants or genomic loci that are associated with SCD. These include variants in the cardiac ion channels KCNQ1 and SCN5A [7], nitric oxide synthase 1 adaptor protein [8], and a susceptibility locus at 21q21 for ventricular fibrillation in patients who have had acute myocardial infarction [9]. Furthermore, common variants in at least 10 genomic loci have been correlated with QT duration, a key indicator of cardiac repolarization [10], [11].

While considerable research has been directed to the identification of the genomics of life threatening arrhythmias (LTA), there has not yet been a genome-wide assessment of patients who have received an implantable cardioverter-defibrillator (ICD). ICDs are implanted in approximately 250,000 individuals in the United States annually for criteria that include diminished ejection fraction, symptomatic heart failure, and to a lesser extent, prolongation of the QRS interval or other primary arrhythmogenic cardiomyopathies. While ICDs have a success rate of more than 97% for sensing and terminating the LTA [12], they are never activated in approximately 80% of patients over the duration of their lives [13]–[15]. Accordingly, our current criteria for selecting patients for ICD therapy are rather crude, particularly when one considers up-front cost approaching $30,000 and the risk, albeit small, of infection, lead and device malfunctions, and inappropriate shocks. At the same time, many patients who could benefit from an ICD do not receive one. Therefore, there is a need for better approaches to risk stratification.

The hypothesis of the current study was that a genome-wide assessment of patients with ICDs would identify common DNA sequence variants associated with LTA and would refine ICD selection criteria. Furthermore, by better defining the population that could benefit from ICD therapy, the information might be extrapolated to identify individuals at risk in the general population who do not currently meet guidelines for primary prevention ICD therapy.

We present the results of a retrospective analysis on patients with an ICD and extended follow-up who had experienced LTA, with a cumulative 607 cases and 297 controls included in the analysis. We genotyped DNA samples using the Illumina Human660 W Genotyping BeadChip and tested for association between genotype at common variants and the phenotype of having an LTA.

## Methods

### Ethics Statement

The Scripps Institutional Review Board reviewed and approved the protocol entitled, MEDTRONIC GAME: Genetic Arrhythmia Markers for Early Detection, IRB #08-4985, on June 6, 2008. The protocol underwent continuing review on May 29, 2009 and was closed with a Final Report on July 22, 2009. Written informed consent was obtained from all participants involved in this study. A copy of the last approved informed consent form is included as Appendix S2.

Barbara G. Bigby, ALM, CIP, Scripps IRB Officer

### Patient Population

**Figure 1:**
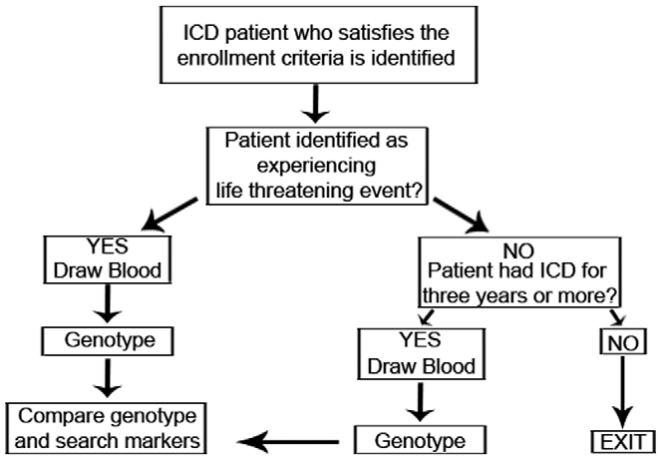
Study design for genome-wide association study of ICD activation with LTA.

The overall study design is shown in Figure 1. The inclusion criteria for all patients was that an ICD or cardiac resynchronization therapy with defibrillator (CRT-D) was implanted, that the patient was considered to be on optimal medical therapy by the treating physician, that coronary artery disease was present with either prior myocardial infarction, percutaneous coronary intervention or coronary artery bypass surgery, and that the patient was of European ancestry, with both parents and all 4 grandparents self-reported as Caucasian. To qualify as cases, the additional inclusion criteria were (1) age >40 years at the time of ICD implantation, and (2) at least one fully documented life threatening arrhythmia, as defined by a spontaneous ventricular arrhythmia with cycle length ≤400 ms appropriately treated by the device. Two hundred of the 607 case patients were secondary prevention patients. Although all patients in the study were expected to have a myocardial infarction (MI) or coronary artery disease (CAD), it was not required that the MI/CAD had to be diagnosed prior to the arrhythmic event date for the secondary prevention patients. To qualify as controls, patients required at least 3 years of ICD therapy with a center-verified absence of any appropriately treated life-threatening arrhythmias and no pre-implantation history of LTA or SCA (i.e. a primary-prevention ICD indication). All control patients all had ICDs for primary prevention. The control data were treated as right censoring at exam date. The demographic data from cases and controls were compared using a t-test.

Patients were recruited at 34 sites across the United States (Appendix S1) and the local institutional review boards approved the protocol. A cohort of 500 cases and 500 controls was targeted for enrollment in keeping with the success of the genome-wide association studies that had a similar sample size in related cardiovascular phenotypes of coronary heart disease [16], atrial fibrillation [17], and QT interval [18]. All patients provided informed consent prior to enrollment.

### DNA isolation

All patients had a single tube of blood drawn at the time of enrollment, which was collected centrally for subsequent DNA extraction. A 0.2 ml aliquot of whole blood from each participant was used for DNA isolation using the Qiagen QIAamp DNA Mini Kit (Qiagen, Valencia, CA; Catalog #51185) and QiaCube Robotic workstation for automated DNA purification. The typical yield was 2–10 ug DNA from 0.2 ml blood. DNA concentrations were determined using a nanodrop spectrophotometer and DNA concentrations were adjusted to 50 ng/ul.

### Genotyping and Data Quality Control

Genotyping was performed using the Illumina 660 W BeadChip, following the manufacturer's instructions. Genotypes were clustered within GenomeStudio using all samples with >98% call rates. SNPs with call rates <95%, or heterozygote frequencies >65% after re-clustering were removed. Cluster separation scores were generated using Illumina's Genome Studio software. After visual inspection of the range of cluster separation scores in this dataset, thresholds for filtering were determined. Autosomal SNPs with cluster separation scores ≤0.30 and X-chromosome SNPs with cluster separation scores ≤0.38 were removed. Sample call rates were recalculated and individuals with call rates <99% were removed (median call rate = 99.989%). Genotype concordance was calculated based on 12 duplicate samples (99.998%).

Additional quality control was undertaken using the genetic analysis program PLINK [19]. Individuals were tested for gender consistency, cryptic relatedness, and ancestry. All genders determined by genotype data agreed with the clinical data. Cryptic relatedness was tested using the –genome command, which was run on SNPs that had been filtered for linkage equilibrium (N = 105,837, r^2^<0.5 within a window of 50 SNPs). Based on the proportion of relatedness (PI_HAT), 3 pairs of samples were duplicate or twin samples (PI_HAT ∼1) and 1 pair of siblings (PI_HAT ∼0.5) were present. In these cases, one individual from each related pair was removed. Samples were clustered by multidimensional scaling with HapMap III samples using the set of 105,837 SNPs in linkage equilibrium. There were 4 samples that clustered outside the European (CEU and TSI) ancestry groups and were removed. A total of 904 samples passed all quality control filters, consisting of 607 cases, 297 controls.

SNPs were additionally filtered for minor allele frequency (>0.01 in all samples) and Hardy-Weinberg equilibrium (P<10^−6^ in the controls). Finally, if SNPs were not present in dbSNP or did not map uniquely to the reference genome, they were removed (N = 645). A total of 534,690 genotyped SNPs passed all quality control filters.

### Genotype Imputation

We imputed genotypes in genotyped individuals for all HapMap (phase II, release 22) SNPs using the program MaCH [20] and an r^2^ threshold of 0.3. The best estimate of the quantitative allele dosage was used as the predictor in association tests. The CEU HapMap phased haplotypes were used as a reference (N = 60 unrelated individuals). We estimated error rates by masking 1% of the genotypes: genome-wide, the allelic error rate was 0.017 and the genotypic error rate was 0.034.

### Association

Case-control association was performed using logistic regression in PLINK. For SNPs that were directly genotyped, the genotype calls were used, while MaCH maximum likelihood dosages (0–2) were used for SNPs that were imputed. Genetic background was estimated using multidimensional scaling (MDS) and the top 10 MDS components were tested for association with the phenotype [21]. Q-Q plots and genomic inflation factors were calculated in PLINK using the –qq-plot command. The survival analysis was performed using a Cox proportional hazards model as implemented in the survival [22], [23]. For the subset of cases with length of follow-up (N = 258), the time between implant and having an adjudicated LTA was used, while for controls (N = 297), the length of follow-up was the time between implant date and exam date. Gender was included as a covariate.

### Power

Power was calculated for a range of allele frequencies and genotyping relative risks based on sample size of cases and controls, minor allele frequency, and genotyping relative risk assuming an additive genetic model. Power at each genotypic relative risk was calculated for an alpha of 5×10^−8^ with CaTS [24].

### CNV analysis

Copy number variants (CNVs) were predicted based on intensity and genotype information from CNV and SNP probes using PennCNV [25] (2010May01 version) and QuantiSNP [26] (v2.3) with default parameters. A total of 33 samples were excluded for high log R Ratio standard deviation, high waviness factor, or drifting B allele frequency. CNVs covering at least 10 probes and with a length of at least 100 kb were obtained using each method. Regions that overlapped between the methods were merged and analyses were performed using CNVs predicted by either method. Only regions that overlapped between the methods were used in the final analysis. There were 4,467 total autosomal CNV regions consisting of 2,193 deletions and 2,274 duplications. Association between cases and controls was run using the rare CNV commands in PLINK with 100,000 permutations. Three analyses were run: all CNVs, deletions (copy number = 0 or 1), and duplications (copy number = 3, 4, or 5). P-values were calculated using a max(T) permutation using the –mperm function in PLINK [19]. This test compares each observed test statistic against the maximum of all permuted statistics over all SNPs for each single replicate. Regions with permuted P-value of less than 0.05 are reported.

## Results

The baseline demographics are summarized in Table 1, the electrophysiologic features at baseline in Table 2, and medications in Table 3.

**Table 1:** Demographics of Cases and Controls at Time of ICD Implantation.

Patient Characteristics	Case Arm (N = 607)	Control Arm (N = 297)	p-value
Age at Visit	71.1±10.0	77.9±5.8	<0.0001**
Gender (Male)	474 (89%)	230 (84%)	0.059
Most Recent LVEF	34.3±12.9	36.7±13.0	0.012*
LVEF Not Available	43 (7%)	22 (7%)	0.891
Weight (Kilograms)	90.1±19.1	85.2±15.9	<0.0001**
Height (Meters)	1.76±0.09	1.74±0.09	0.010*
Body Mass Index	29.1±5.4	28.0±4.8	0.006**
RV - MI	44 (7%)	23 (8%)	0.788
LVA - MI	143 (24%)	89 (30%)	0.043*
LVP - MI	72 (12%)	19 (6%)	0.010*
Septal MI	37 (6%)	27 (9%)	0.128
Unknown MI Location	264 (43%)	118 (40%)	0.316
No Documented MI	100 (16%)	55 (19%)	0.453
Approximate Age at First MI	55.5±11.6	60.9±10.8	<0.0001**
Family History of Sudden Cardiac Arrest or Death	148 (37%)	85 (43%)	0.154
Earliest Age of SCA/SCD for Parents/Siblings	61.7±13.3	64.8±13.5	0.115

**Table 2:** Electrophysiological characteristics of cases and controls.

Electrophysiological Characteristics	Case Arm (N = 607)	Control Arm (N = 297)	p-value
QRS> = 120 ms	218 (54%)	122 (66%)	0.005**
Most Recent Intrinsic QRS Duration	128.4±35.8	138.0±39.8	0.004**
QRS Duration Not Available	203 (33%)	113 (38%)	0.182
Atrial Fibrillation	250 (41%)	145 (49%)	0.032*
Atrial Flutter	59 (10%)	30 (10%)	0.906
Atrial Tachycardia	24 (4%)	7 (2%)	0.248
AV Block	49 (8%)	53 (18%)	<0.0001**
AV Nodal Re-Entrant Tachycardia	4 (1%)	0 (0%)	0.309
AV Re-Entrant Tachycardia	1 (0%)	1 (0%)	0.549
Familial or Inherited Conditions with High Risk for VT	10 (2%)	1 (0%)	0.113
Intraventricular Conduction Delay	14 (2%)	13 (4%)	0.097
Left Bundle Branch Block	67 (11%)	50 (17%)	0.020*
Right Bundle Branch Block	38 (6%)	23 (8%)	0.400

**Table 3:** Baseline use of key medications.

Medication Use	Case Arm (N = 607)	Control Arm (N = 297)	p-value
ACE Inhibitor Use - Current	294 (61%)	143 (58%)	0.426
ACE Inhibitor Use - Ever	444 (92%)	216 (87%)	0.046*
Angiotensin Receptor Blocker Use - Current	117 (36%)	57 (31%)	0.286
Angiotensin Receptor Blocker Use - Ever	174 (53%)	85 (46%)	0.119
Aldosterone Antagonist Use - Current	81 (27%)	31 (17%)	0.019*
Aldosterone Antagonist Use - Ever	130 (43%)	49 (27%)	0.001**
Beta Blocker Use - Current	467 (82%)	229 (82%)	1.000
Beta Blocker Use - Ever	559 (98%)	274 (98%)	0.800
Diuretics Use - Current	362 (74%)	188 (75%)	1.000
Diuretics Use - Ever	445 (92%)	223 (88%)	0.187
Anti-Arrhythmic/Class I Use - Current	44 (16%)	10 (6%)	0.002**
Anti-Arrhythmic/Class I Use - Ever	81 (29%)	19 (11%)	<0.0001**
Anti-Arrhythmic/Class II Use - Current	34 (13%)	16 (10%)	0.283
Anti-Arrhythmic/Class II Use - Ever	56 (22%)	16 (10%)	0.001**
Anti-Arrhythmic/Class III Use - Current	167 (42%)	36 (18%)	<0.0001**
Anti-Arrhythmic/Class III Use - Ever	259 (65%)	68 (34%)	<0.0001**

Cases and controls differed by age, height, weight, location of myocardial infarction, and age of first myocardial infarction (Table 1). In addition, there were 704 males and 200 females in the study. Gender specific differences were observed in mean left ventricular ejection fraction (LVEF), weight, height, smoking, and approximate age for the first MI.

The electrophysiologic characteristics at baseline also differed between the groups with respect to incidence of atrioventricular block, left bundle branch block, and a history of spontaneous ventricular arrhythmia (Table 2). For the 102 patients with qualifying tachycardias, the mean cycle length was 297 msec (S.D. = 56.5). The histogram of average ventricular cycle length for these 102 patients is shown in Figure S1. Finally, there were also some imbalances in baseline medications, particularly anti-arrhythmic drugs (Table 3).

**Figure 2:**
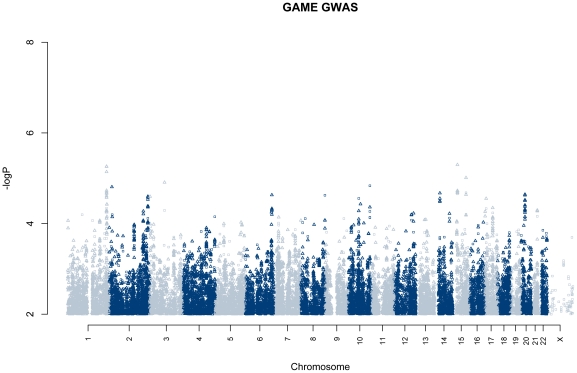
Genome-wide association results of Cases with LTA vs. Controls without a LTA. Association −log(P-values) are plotted according to position in the genome. Points indicate genotyped (circle) or imputed (triangle) SNPs.

A genome-wide association was performed between individuals that had an LTA (cases) and those that did not (controls) over the course of at least three years (Figure 2). Gender and genetic background were tested for association with case-control status, were not found to be significant, and were not included in the model. We did not detect any regions associated at P<5×10^−8^ and report top regions at P<1×10^−5^ (Table 4). The strongest association at rs11856574 (P = 5.0×10^−6^), located in the hypothetical gene *KIAA0574* (hypothetical protein LOC23359). We detected no evidence of population stratification (genomic inflation factor = 1.0035, Figure S2). On a subset of individuals for which we had length of follow-up, we performed survival analysis GWA (Figure S3). We did not detect any associations at P<5×10^−8^, but do note a region at 13q14.2 (rs2854357 P = 8.2×10^−^7) with a consistent signal across multiple SNPs.

**Table 4:** Top regions associated at P<10^−5^.

Top SNP	Alleles (A1/A2)	Chr	Pos	#SNPs at P<10^−4^	Freq (A1)	OR (A1)	P	Nearest gene, description, relative SNP location
rs11856574*	G/A	15	27518736	1/13	0.86	2.02	5.0×10^−6^	*KIAA0574*, hypothetical protein, intron
rs482329*	C/G	1	232883177	3/18	0.61	1.60	5.5×10^−6^	*IRF2BP2*, interferon regulatory factor 2 binding protein 2, 72 kb downstream
rs3848198*	C/T	15	78426619	2/4	0.38	1.81	9.8×10^−6^	*ARNT2*, Aryl hydrocarbon receptor nuclear translocator 2; hypoxia associated transcription factor, 57 kb upstream
rs6565373*	T/C	16	86817543	0/1	0.59	0.32	9.8×10^−6^	*BANP*, BTG3 associated nuclear protein isoform a; negative regulator of p53 transcription, 149 kb downstream

*imputed; #SNPs at P<10^−4^ indicates the number of SNPs at P<10^−4^ within +/−150 kb of the top SNP that were either directly genotyped or imputed (genotyped/imputed).

We looked specifically at P-values for 42 SNPs previously reported to be implicated in prolonged QT duration or SCD [10], [11], [17], [18], [27]–[30]. None were associated in the study dataset at Bonferroni corrected P-values (P<0.05/42 = 0.0012). Table S1 shows the P-values from directly genotyped or imputed genotypes for these 42 SNPs, indicating that these SNPs may be of limited prognostic value in identifying individuals likely to have an LTA among those that are candidates for an ICD with prior myocardial infarction.

We calculated our power to detect effects at an alpha of 10^−7^ for the sample sizes observed here. We were well powered to detect effects of large effect size (OR>3.78) in common variants (minor allele frequency (MAF)>0.05) given our sample size (Figure S4). For very common variants (MAF>0.25), we had 80% power to detect effects of OR>2.15. Thus, it is unlikely that there is a common variant with a large effect strongly associated with LTA in individuals of European ancestry. Using genome-wide genotyping chips such as the Ilumina Human660 BeadChip, there is good coverage of common variation in European ancestry populations [31], however not all regions of the genome, in particular regions of low linkage disequilibrium, may be covered well.

We conducted a copy number variation analysis, using QuantiSNP and PennCNV to estimate copy number variation in each individual. CNV regions covering at least 10 probes and 100 kb from both methods were combined and tested for association with case/control status in PLINK. There were no regions that were associated at a multiple testing corrected P-value less than 0.05 when all CNVs were combined. However, one region on chromosome 16 (position 33,395,681–33,506,617) was associated (minimum P = 0.0097) when deletions alone were tested (Figure S5). The region is flanked by a target of p53 (*TP53TG3*) and a creatine transporter (*SLC6A8*). As this region is a duplication of a region on chromosome X [32], we tested whether having a CNV in this region was associated with gender and saw no association. These CNVs require validation using quantitative PCR and the association will require verification in a replication study.

## Discussion

Although ICDs are the main therapy for preventing SCD, current criteria for patient selection remains suboptimal with only approximately 20% requiring appropriate therapy over their lifetime [13]–[15]. Through a genome-wide assessment, we were unable to identify any common sequence variants that were associated with appropriate ICD therapy for a LTA. We did detect a marginally significant deletion associated with case status, but this will require further validation and replication before it can be considered a positive finding.

Our sample size was adequate to detect common sequence variants that confer large effects with minor allele frequency of 0.25 or greater, many of which have previously been described to be genome-wide associated with prolonged QT duration or in candidate gene studies of SCD [6]–[11], [17], [18], [27]–[30]. There are several potential explanations for our findings. First, it is possible that low frequency or rare sequence variants are associated with appropriate ICD therapy. Indeed, several recent studies have demonstrated that for common clinical phenotypes, multiple rare variants with high penetrance may play an important role in accounting for heritability [33]–[35]. It should be pointed out that we screened common SNPs and copy number variants, but our methodology did not address the detection of other sequence variants such as small insertions, small deletions, or other forms of structural genomic variation. Second, there may be relatively common SNPs that confer a smaller effect (OR<2.0), but were not detected in our cohort due to inadequate statistical power or due to combining all types of LTA with rates ≤400 msec. This potential shortcoming could be overcome by combining the data from multiple like cohorts to determine whether gene variants with minor allele frequency between 5% and 25% may, at least in part, contribute to the heritability of LTA. Third, it remains possible that propensity to develop an LTA is not genetically determined, particularly in the types of patients who currently receive ICDs. Such patients have markedly reduced systolic left ventricular function. The likelihood of having appropriate ICD therapy increases over time [36], and may be related to unfavorable cardiac remodeling and adrenergic activation, representing more of a mechanical/neural substrate than one that would implicate an intrinsic abnormality of ion channels, repolarization, or electrical dispersion. This possible explanation appears to be offset to some degree by the remarkable finding of a common variant at 21q21 for ventricular fibrillation in patients with acute myocardial infarction [9], although the arrhythmogenic milieu of acute myocardial infarction differs from that of SCA in the stable patient of systolic dysfunction. Fourth, it is possible that our cohort was quite heterogeneous or suboptimal from a number of standpoints. The lack of more extended follow-up may have led to misclassification of phenotype, or the multiple paths to low ejection fraction (e.g. myocardial infarction, diffuse coronary artery disease cardiomyopathy) might represent a mosaic with inadvertent lumping of phenotypes and diminished ability to discern the genomic factors associated with any given subtype. There is some indication from the subgroup of patient with extended follow up in our study that this may represent a key issue for detection of a significant genomic association.

It is worth noting that the study cohort is representative of the demographics for patients in the United States who receive an ICD. Patients who are in the control arm were slightly older, likely because of the selection bias requiring that the individuals were event-free and followed for a minimum of three years following the device implant.

ICDs have been shown to reduce all-cause mortality by 26% and 57% reduction of SCD in randomized controlled trials [36]. Observational data from 11 case-control cohorts involving 96,951 patients has been even more impressive with a 46% reduction of all-cause mortality [36]. Yet the current selection criteria for ICD implantation are far from optimal. About 80% of patients do not require ICD activation for a life-threatening arrhythmia during their lifetime after implantation. This lack of discriminative ability is especially problematic given the cost of the devices and the array of complications, albeit at low incidence, but including implantation-related complications, lead failures, device malfunction, and inappropriate shocks.

Moreover, a large proportion of patients with SCD do not meet any current criteria for ICD therapy [1]. They have intact ejection fraction and, at present, have no identifying risk markers. Had genomic loci become evident from the present study of a cohort with ICDs, it remains possible that the findings would be informative or extrapolatable to the general population. With the negative findings here, it remains unclear whether the ICD cohort will prove to be representative of the at-large population with risk of SCD.

There have been a very large number of genome-wide association studies performed, and a subset of these have been published for over 150 complex traits [37]. The negative GWAS for LTA represents an important finding with the bias of publication of only positive results. Here we have attempted to identify genomic markers that would refine our appropriate selection of patients who might receive an ICD, above and beyond those patients with significant left-ventricular dysfunction. Our inability to demonstrate a genomic marker should not be considered an abject failure, but rather it can be viewed as the first step that lays the groundwork for more intensive efforts such as deep sequencing of candidate genes or much larger cohorts with more extended follow-up for genome-wide association. This area of medicine, exemplifying device genomics, and especially at a time of crisis in health care economics, deserves further study.

## Supporting Information

Figure S1 **Histogram of average ventricular cycle length for subjects with qualifying tachycardia event (N = 102).**
(JPG)

Figure S2 **Q-Q plot of LTA association.** −log(P-values) are plotted according to expected (x-axis) and observed (y-axis) values. Expected values were calculated in PLINK. Only directly genotyped SNPs were used in this plot. A line is drawn at y = x.
(JPG)

Figure S3 **Survival analysis Manhattan Plot.** Genome-wide association −log(P-values) are shown for a survival analysis using individuals (258 cases, 297 controls) for which we had length of follow-up. Points indicate genotyped (circle) or imputed (triangle) SNPs.
(JPG)

Figure S4 **GWAS power.** Genotypic relative risk (GRR) detected at 80% power with at alpha = 5×10^−8^ for 607 cases and 297 controls is plotted as a function of minor allele frequency.
(JPG)

Figure S5 **Deletions overlapping chr 16 CNV association.** CNVs overlapping at least 10 probes and 100 kb were predicted using PennCNV and QuantiSNP. CNVs predicted in controls (blue) and cases (red) are plotted by location on the chromosome. SNP and CNV markers are shown as asterisks or triangles, respectively and plotted by location.
(JPG)

Table S1 **Association at SNPs previously implicated in sudden cardiac death, prolonged QT duration, atrial fibrillation, or ventricular fibrillation.**
(DOC)

Appendix S1 **Enrolling Center List.**
(PDF)

Appendix S2 **IRB Approved** ***Consent to Participate in Research*** **Form.**
(PDF)
