# Supplementary figures and images for: Genome-Wide Association of Implantable Cardioverter-Defibrillator Activation With Life-Threatening Arrhythmias
Source: PLoS One. 2012 Jan 11;7(1):e25387. doi: 10.1371/journal.pone.0025387 (PMC3256134; doi:10.1371/journal.pone.0025387)

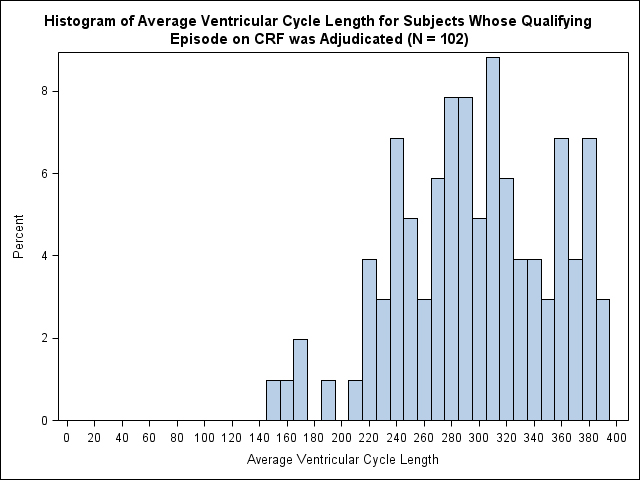

Supplement: Figure S1 — Histogram of average ventricular cycle length for subjects with qualifying tachycardia event (N = 102). (JPG) [file pone.0025387.s001.jpg]

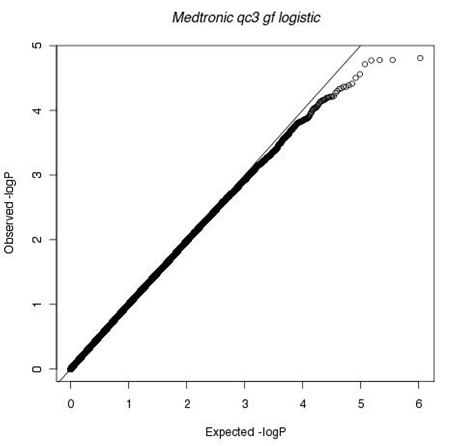

Supplement: Figure S2 — Q-Q plot of LTA association. −log(P-values) are plotted according to expected (x-axis) and observed (y-axis) values. Expected values were calculated in PLINK. Only directly genotyped SNPs were used in this plot. A line is drawn at y = x. (JPG) [file pone.0025387.s002.jpg]

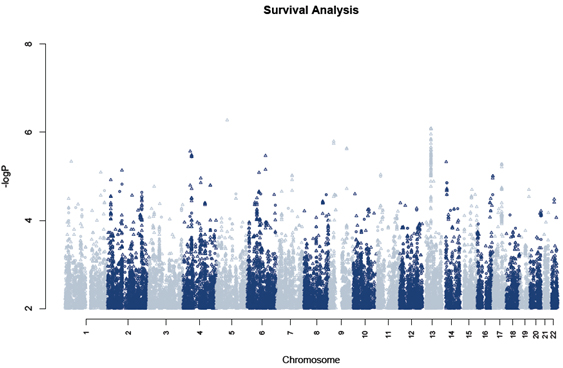

Supplement: Figure S3 — Survival analysis Manhattan Plot. Genome-wide association −log(P-values) are shown for a survival analysis using individuals (258 cases, 297 controls) for which we had length of follow-up. Points indicate genotyped (circle) or imputed (triangle) SNPs. (JPG) [file pone.0025387.s003.jpg]

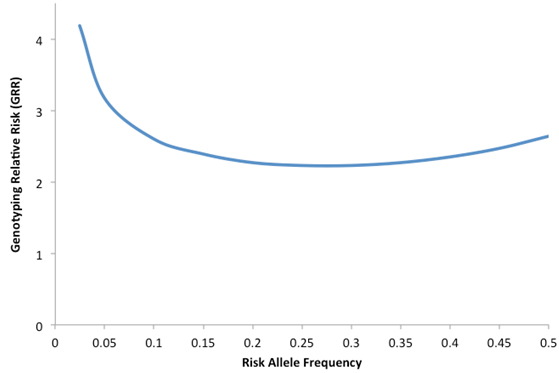

Supplement: Figure S4 — GWAS power. Genotypic relative risk (GRR) detected at 80% power with at alpha = 5×10^−8^ for 607 cases and 297 controls is plotted as a function of minor allele frequency. (JPG) [file pone.0025387.s004.jpg]

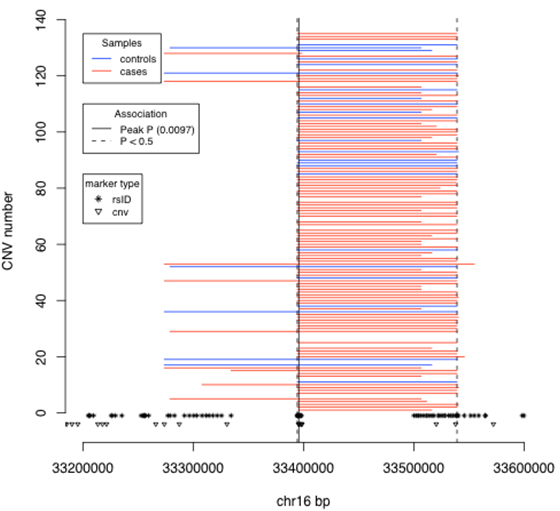

Supplement: Figure S5 — Deletions overlapping chr 16 CNV association. CNVs overlapping at least 10 probes and 100 kb were predicted using PennCNV and QuantiSNP. CNVs predicted in controls (blue) and cases (red) are plotted by location on the chromosome. SNP and CNV markers are shown as asterisks or triangles, respectively and plotted by location. (JPG) [file pone.0025387.s005.jpg]
