# Supplementary material for: Genome-Wide Association of Implantable Cardioverter-Defibrillator Activation With Life-Threatening Arrhythmias
Source: PLoS One. 2012 Jan 11;7(1):e25387. doi: 10.1371/journal.pone.0025387 (PMC3256134; doi:10.1371/journal.pone.0025387)
Supplement: Table S1 — Association at SNPs previously implicated in sudden cardiac death, prolonged QT duration, atrial fibrillation, or ventricular fibrillation. (DOC) [file pone.0025387.s006.doc]

| **Supp Table 1: Association at SNPs previously implicated in sudden cardiac death, prolonged QT duration, atrial fibrillation, or ventricular fibrillation ^(references from manuscript)^** | | | | | | |
| --- | --- | --- | --- | --- | --- | --- |
| SNP | Gene/Region | A1/A2 | Freq A1 | OR | P | Reference/Trait |
| rs7692808 | *ARHGAP24* | A/G | 0.29 | 0.98 | 0.833 | PR interval^30^ |
| rs10919071 | *ATP1B1* | A/G | 0.87 | 0.95 | 0.709 | QT interval^11^ |
| rs7341478 | *CACNA2D1* | A/G | 0.27 | 0.94 | 0.611 | QT interval^18^ |
| rs3807989 | *CAV1/CAV2* | A/G | 0.40 | 0.88 | 0.201 | PR interval, AF^29,30^ |
| rs37062 | *NDRG4* | A/G | 0.76 | 0.90 | 0.380 | QT interval^10^ |
| rs1733724 | *DKK1* | G/A | 0.75 | 1.33 | 0.013 | PR interval^29^ |
| rs6585682 | *FGFR2* | C/T | 0.47 | 1.02 | 0.843 | QT interval^18^ |
| rs3804999 | *ITPR1* | A/G | 0.71 | 1.26 | 0.036 | QT interval^18^ |
| rs1805128 | *KCNE1* | C/T | 0.99 | 1.09 | 0.842 | QT interval^10^ |
| rs2968863 | *KCNH2* | C/T | 0.76 | 0.96 | 0.754 | QT interval^10,11^ |
| rs2968864 | *KCNH2* | T/C | 0.76 | 0.97 | 0.772 | QT interval^10^ |
| rs17779747 | *KCNJ2* | G/T | 0.67 | 0.92 | 0.445 | QT interval^11^ |
| rs2282428 | *KCNK1* | C/T | 0.35 | 0.95 | 0.646 |  |
| rs13376333 | *KCNN3* | C/T | 0.69 | 0.92 | 0.452 | Atrial Fibrillation^28^ |
| rs12576239 | *KCNQ1* | C/T | 0.87 | 1.36 | 0.041 | QT interval^10^ |
| rs12296050 | *KCNQ1* | C/T | 0.82 | 1.27 | 0.065 | QT interval^11^ |
| rs2074328 | *KCNQ1* | C/T | 0.97 | 0.91 | 0.746 | QT interval^10^ |
| rs2074518 | *LIG3* | T/C | 0.49 | 1.07 | 0.495 | QT interval^10^ |
| rs8049607 | *LITAF* | T/C | 0.49 | 0.94 | 0.539 | QT interval^10,11^ |
| rs11897119 | *MEIS1* | C/T | 0.40 | 1.04 | 0.676 | PR interval^30^ |
| rs365990 | *MYH6* | G/A | 0.37 | 1.02 | 0.853 | Heart Rate^29^ |
| rs7188697 | *NDRG4* | A/G | 0.75 | 0.94 | 0.614 | QT interval^11^ |
| rs251253 | *NKX2-5* | T/C | 0.62 | 1.03 | 0.826 | Atrial Fibrillation, PR interval^30^ |
| rs12029454 | *NOS1AP* | G/A | 0.85 | 1.08 | 0.575 | QT interval^10^ |
| rs12143842 | *NOS1AP* | C/T | 0.76 | 1.06 | 0.599 | QT interval^10^ |
| rs16857031 | *NOS1AP* | C/G | 0.87 | 0.97 | 0.844 | QT interval^10^ |
| rs2200733 | *PITX2* | C/T | 0.88 | 0.87 | 0.378 | Atrial Fibrillation^17^ |
| rs6843082 | *PITX2* | A/G | 0.78 | 0.95 | 0.698 | Atrial Fibrillation^28^ |
| rs10033464 | *PITX2* | G/T | 0.90 | 1.04 | 0.825 | Atrial Fibrillation^17^ |
| rs11970286 | *PLN* | T/C | 0.45 | 0.95 | 0.597 | QT interval^11^ |
| rs7146384 | *QTC_14.1* | G/A | 0.67 | 1.20 | 0.070 | QT interval^18^ |
| rs1559578 | *QTC_5.3* | T/C | 0.65 | 1.05 | 0.649 | QT interval^18^ |
| rs846111 | *RNF207* | G/C | 0.72 | 0.97 | 0.844 | QT interval^10,11^ |
| rs6795970 | *SCN10A* | A/G | 0.38 | 0.86 | 0.142 | PR interval^29^, Pacemaker^29^, VF^27^ |
| rs11708996 | *SCN5A* | G/C | 0.85 | 0.82 | 0.181 | PR interval^30^ |
| rs11129795 | *SCN5A* | G/A | 0.75 | 0.91 | 0.450 | PR interval^29^, QT interval^11^ |
| rs12053903 | *SCN5A* | T/C | 0.66 | 1.07 | 0.520 | PR interval^29^, QT interval^11^ |
| rs11047543 | *SOX5* | G/A | 0.86 | 0.98 | 0.911 | PR interval, Atrial Fibrillation^30^ |
| rs13038095 | *SULF2* | G/T | 0.90 | 0.98 | 0.929 | Atrial Fibrillation^28^ |
| rs3825214 | *TBX5* | A/G | 0.80 | 1.05 | 0.672 | QRS duration, Atrial Fibrillation^29^ |
| rs1896312 | *TBX5-TBX3* | T/C | 0.74 | 1.08 | 0.515 | PR interval^30^ |
| rs4944092 | *WNT11* | G/A | 0.32 | 1.11 | 0.334 | PR interval^30^ |
