# Supplementary material for: Genome-Wide Association of Implantable Cardioverter-Defibrillator Activation With Life-Threatening Arrhythmias
Source: PLoS One. 2012 Jan 11;7(1):e25387. doi: 10.1371/journal.pone.0025387 (PMC3256134; doi:10.1371/journal.pone.0025387)
Supplement: Appendix S1 — Enrolling Center List. (PDF) [file pone.0025387.s007.pdf]

Appendix A: Enrolling Center List

Clinic	Physician
Amarillo	Abdallah
Arkansas	Norris
Baylor	Kowal
Baylor-Plano	DeVille
Cardio Consultants	Hodgkin
Central Coast	Grogin
Chattanooga	Bruce
Desert Cardio	Rubin
Eastlake	Sayar
Emory	Langberg
Glendale	McKenzie
Hall-Garcia	Massumi
Harris	McBride
Heart Center Florida	Sayad
Heart Clinic Arkansas	Chakka
Huntington	Maccaro
Indiana Heart	Genovely
Inland	Alaeddini
Krannert	Das
Lindner	Kereiakes
Louisville	Imburgia
Mid America	Ramza
NorthShore	Jadoneth
Richmond	Han
Scripps	Rogers
South Carolina	Gottipaty
Spokane	Goldberg
St Paul Heart	Adler
Stern	Mc Grew
Texas	Canby
U of KS	Emert
U of Washington	Rho
Umass	Rosenthal
Willowbrook	Nasir
