# Supplementary material for: Genome-Wide Association of Implantable Cardioverter-Defibrillator Activation With Life-Threatening Arrhythmias
Source: PLoS One. 2012 Jan 11;7(1):e25387. doi: 10.1371/journal.pone.0025387 (PMC3256134; doi:10.1371/journal.pone.0025387)
Supplement: Appendix S2 — IRB Approved Consent to Participate in Research Form. (PDF) [file pone.0025387.s008.pdf]

VOID

CONSENT TO PARTICIPATE IN RESEARCH

Genetic Arrhythmia Markers for Early detection

[GAME study]

Principal Investigator: **John Rogers M.D.**

Sub-Investigators: **Doug Gibson M.D.**
Tom Ahern M.D.
Laura Ornowski R.N., A.N.P.-C

Study Coordinator: Dana Campbell R.N., B.S.N., C.C.R.P.
(858) 554-5751

Research Site(s): **Scripps Clinic, Torrey Pines**
Scripps Green Hospital
Scripps Translational Science Institute and Genomic Medicine
The Scripps Research Institute (TSRI)
10666 N. Torrey Pines Road SW206
La Jolla, CA 92037

Sponsor: **Medtronic, Inc.**

Before you start reading about this research, please read the California Experimental Subjects' Bill of Rights, which is located on page 7 of this document.

Why is this research being done?
You have been asked to participate in the GAME study because you have an Implantable Cardioverter Defibrillator (ICD) device.

The purpose of the GAME study is to develop a new blood test that may allow doctors to better identify people who might benefit from ICD devices.

This study is sponsored by Medtronic, Inc. About 2,000 subjects may participate in this study at up to 50 centers in the United States, with up to 200 of them here at Scripps.

In this study, researchers will analyze information about your genes from your blood samples. Genetic information comes from DNA found in cells. Differences in genetics account for many differences between people, like eye color or height. You receive genes from both of your parents. They inherited their genes from their parents. Family members share genes.

Some genes may be associated with illnesses or disease. These diseases are caused by defective genes that make proteins that don't work correctly. Some genes may make it more likely that one person will

get sick. Doctors and scientists are learning more every day about how genes contribute to health and illness. Researchers study genes from people affected by various diseases to learn which genes or forms of genes might be associated with certain diseases.

How long is the study?
If you agree to join, you will be in the study for 1 – 2 days.

What will happen to me?
Your participation in this study will involve one blood draw, followed by an optional 24 hour Holter monitor, which is a portable ECG recorder that consists of a small cassette-like box that is worn around the chest or the waist.

Blood sample:
If you agree to take part in this study, you will be asked for a blood sample to be taken from your arm in the same way as any previous blood test you may have had. The nurse or lab technician will insert a needle into your vein to draw out the blood. One tube of blood (about two teaspoons) will be collected.

Medical Information collected:
Genetic information from the blood sample will be compared with other health information collected from you as a part of the GAME study. This other health information will be collected through an interview and physical examination at the time of enrollment or through review of your medical records. This information includes your medical history, symptoms and side effects, exam results, blood tests, medications, and information on your ICD.

Holter monitoring:
You may be asked to wear a Holter recorder for up to a 24 hour period. The Holter recorder is connected to your body with electrodes (sticky patches) and a soft, small, flat, donut-shaped antenna is taped to the skin over your ICD. The clinic staff will hook up the Holter monitor and make sure that it is working properly.

You will wear the monitor for 24 hours. After that, you will be asked to return to the clinic to have the monitor removed. If you prefer, you can remove the Holter monitor at home and return it using a pre-addressed, pre-paid mailing envelope. Information from the Holter monitor will be analyzed by Medtronic, the sponsor of this study.

This information is useful because it may help researchers develop methods to predict which patients are at risk of developing life threatening heart rhythms.

Could I experience any side effects or discomforts?
Blood sampling:
It may hurt when the needle pokes your skin. You could be sore and bruised for a day or two. You could get an infection, but that's not likely. If you have ever felt dizzy or fainted while having blood drawn, you should tell the person drawing your blood. If you lie down, you might not get dizzy.

Holter monitor:
The risk of the 24 hour Holter monitor recording is low. The tape used to fasten the electrodes and antenna may cause skin irritation in some patients. The potential risks to the subject are believed to be the

VOID

same as those encountered during a standard ECG testing or Holter recording. You will not be allowed to shower or get the monitor wet.

Medical information and confidentiality:
It is possible that analysis of the genetic information from the study blood sample may reveal factors related to medical conditions other than heart conditions. No information will be given to you or your physician about the research test results.

Any risks to your privacy are discussed under "What about Confidentiality?"

Although we have tried to identify all possible risks, there may be risks that are not yet known. You should discuss any questions you have about possible risks and discomforts with your doctor.

Will I benefit from this research?
There will be no direct benefits to you by taking part in this research study.

The information from the study may help patients in the future by developing a blood test that might identify genetic information associated with heart conditions.

Will I get paid?
No, you will not be paid for your participation in this study.

Will it cost anything to be in the study?
No, all testing and services done only for the study will be provided at no cost to you and will not be billed to you or your insurance company. Your standard care will be billed to you or your insurance, as usual.

What if I end the study early?
Participation in the GAME study is entirely voluntary.

If you end the study early, you may be asked to return for tests to be sure your health has not changed during the study.

The investigator may decide to withdraw you from the study at any time for any reason.

If you do withdraw from the study, your study blood sample will be destroyed. All information from testing of the blood sample and health information collected from you in the GAME study up to the time of withdraw will continue to be included in the study.

What other treatments could I take?
This research study does not involve treatment. You can choose not to join.

What are my rights?

- You can call the staff to ask any questions about this study. The telephone number is listed at the top of this form.
- You can decide not to be in this study or you can quit after starting. Whatever you do, your medical care at Scripps will not be affected.

VOID

VOID

- If you have any questions about your rights, call the Scripps Office for the Protection of Research Subjects at (858) 652-5500. You should also read the *Experimental Subject's Bill of Rights,* which is attached to this form.
- You do not have to be in this study. You still have all your legal rights whether you join the study or not.
- You will be told any new information that might make you change your mind about staying in the study.

What are my responsibilities if I join?
If you are in this study, you are expected to:

- Follow the instructions of the research staff
- Report any serious or unusual side effects to the study doctor

What about confidentiality?
If you decide to take part in the study, this form and your study records will be kept separate from your medical record. The sponsor, Medtronic, their agents, representatives, and designees of Medtronic will review your medical records to verify data collected for the study. Regulatory Authorities such as the food and Drug Administration (FDA) and the Institutional Review Board (IRB- a review committee to ensure and review the rights of research subjects) may also inspect your records and this signed consent form to check that the study is being carried out correctly. Because of the need to release information to these parties, they are obligated to keep your data confidential to the extent permitted by the applicable laws and regulations. All appropriate measures will be taken to protect your personal data.

<u>Coding of blood samples and medical information:</u>
The label on your blood sample will be coded with a GAME study identification number at the study site. Blood samples will be sent from the study site to a laboratory outside of Medtronic for analysis and storage. This laboratory will receive only the blood sample with the coded label and will not receive information that will allow them to identify you.

The information produced during analysis of the blood samples at the laboratory will be sent to Medtronic. The information will be coded with GAME study identification numbers. Medtronic will be able to link your GAME study identification number to your health information gathered for the GAME study, so Medtronic (not the laboratory) will have health information that may identify you. However, the study sponsor and the research laboratory commit to make no attempt to identify you as an individual and you will not be contacted further after your participation in the study is complete.

If you are participating in the Holter monitor portion of the study, a special electronic data card will be used to save the recordings. This card will be labeled with the same identification number used for the blood sample. The card will be sent to Medtronic for analysis.

Your health information will be handled as described in this GAME study informed consent form.

For more information, see the **Authorization to use your Private Health Information** at the end of this consent form.

VOID

Is there anything else I should know?

A new Federal law, called the **Genetic Information Nondiscrimination Act (GINA)**, generally makes it illegal for health insurance companies, group health plans, and most employers to discriminate against you based on your genetic information. This law generally will protect you in the following ways:

- Health insurance companies and group health plans may not request your genetic information that we get from this research.
- Health insurance companies and group health plans may not use your genetic information when making decisions regarding your eligibility or premiums.
- This new Federal law ***does not*** protect you against genetic discrimination by companies that sell life insurance, disability insurance, or long-term care insurance.

What if I get hurt in the study?
You can call Dr. John Rogers at (858) 554-5063 and/or the study coordinator Dana Campbell RN at (858) 554-5751 Monday through Friday, 8:00 a.m. to 4:00 p.m. if you get sick or injured while in this study. If you get sick or injured at night or on a weekend, call (858) 455-9100 and ask for the doctor on call for the Division of Cardiology.

If you need medical care as a direct result of being in this study, it will be provided to you. The costs of this treatment will be billed to your health insurance, Medicare, or you. Scripps may ask the sponsor of the study to pay for any costs that are not covered by insurance.

Neither the sponsor nor Scripps plans to provide any compensation for an injury caused by participating in this study.

By agreeing to the above, you are not giving up any of your legal rights by being in this study.

Will Scripps, the study doctor or sponsor benefit from this study?
The study site will receive compensation from the study sponsor (Medtronic, Inc.) for work involved in collecting study data, managing the study at this site, and for procedures being done solely for the study.

Financial Disclosure
Your participation in this study may help the sponsor develop new products from which Medtronic may receive benefit. These products will become the exclusive property of Medtronic, the study sponsor. Medtronic and other researchers may apply for patents on any inventions from this study

If you have any questions about the use of your blood sample, Holter monitoring, or collection of medical information you should ask the study doctor before giving your consent to be in the study. If this research uses your information to make a valuable product, the product will belong to the sponsor or study doctor. There are no plans to share any profits with you.

I agree to participate.

I have read and understood the explanation of the study. The study has also been explained to me by Dr. Rogers or his designee. I have had a chance to ask questions and have them answered to my satisfaction. I agree to take part in this study. I have not been forced or made to feel obligated to take part.

I have read the attached ***Experimental Subject's Bill of Rights*** and the ***Authorization to use my Private Health Information****, which contain some important information about research studies. I must sign this consent form, the* ***Experimental Subject's Bill of Rights*** *and the* ***Authorization to use my Private Health Information****. I will be given a signed copy of each to keep.*

____
Printed Name of Subject

____ ____________________________
Signature of Subject *Date*

____ ____________________________
Signature of person conducting the informed consent discussion Date

____
Role of person named above in the research project

OPTIONAL FUTURE RESEARCH PARTICIPATION

The study sponsor, Medtronic, would like to use the information from the GAME study in future research on the role of genetic and other blood factors in disease and treatments. This includes the study blood sample, genetic information from analysis of the blood sample and health information obtained from the GAME study. Medtronic will establish a process to "de-identify" this research information. This means that no information for future research can identify you.

Please initial below to indicate whether or not you are willing to allow Medtronic to use your information as described here for future research:

________ Yes, I am willing to allow Medtronic to use my information for future research. My blood sample will be kept for up to 20 years strictly for the purpose of research.

________ No, I am not willing to allow Medtronic to use my information for future research and I understand my blood sample will be destroyed following use in the GAME study.

VOID

EXPERIMENTAL SUBJECT'S BILL OF RIGHTS

If I am asked to consent to be a subject in a research study involving a medical experiment, or if I am asked to consent for someone else, I have the right to:

1. *Learn the nature and purpose of the experiment (also called "study" or "clinical trial").*

2. *Receive an explanation of the procedures to be followed in the study, and any drug or device to be used.*

3. *Receive a description of any discomforts and risks that I could experience from the study.*

4. *Receive an explanation of any benefits I might expect from the study.*

5. *Learn about the risks and benefits of any other available procedures, drugs or devices that might be helpful to me.*

6. *Learn what medical treatment will be made available to me if I should be injured as a result of the study.*

7. *Ask any questions about the study or the procedures involved.*

8. *Quit the study at any time, and my decision will not be used as an excuse to withhold necessary medical treatment.*

9. *Receive a copy of the signed and dated consent form.*

10. *Decide to consent or not to consent to a study without feeling forced or obligated.*

If I have questions about a research study, I can call the contact person listed on the consent form. If I have concerns about the research staff, or need more information about my rights as a subject, I can contact the Scripps Office for the Protection of Research Subjects, which protects volunteers in research studies. I may telephone the Office at (858) 652-5500, 8:00 a.m. to 4:00 p.m. weekdays, or I may write to the Scripps Office for the Protection of Research Subjects, Mail Stop SCRC200, 11025 North Torrey Pines Road Suite 200, La Jolla, CA, 92037.

By signing this document, I agree that I have read and received a copy of this Bill of Rights.

____________________________________ ________________
Signature of Subject or Legal Representative Date

**California Health & Safety Code, Section 24172*

VOID

Scripps

Authorization to use your Private Health Information

Name of Study: Genetic Arrhythmia Markers for Early detection (GAME study)

Principal Investigator: John Rogers MD IRB Study Number: 004985

VOID

What is private health information?
Private health information is any information that can be traced back to you. We need your authorization (permission) to use your private health information in this research study. The private health information that we will use and share for this study includes:

- Your past and present health information, including progress notes and history and physicals in your medical record
- Results of your current medical tests and procedures for this study
- Your age, ethnic background and related family history
- Information that can be used to contact you, such as your home address, phone numbers, and emergency contacts if you can not be reached any other way

Who else will see my information?
In addition to the Principal Investigator, this information may be shared with:

- the sponsor of the research study, Medtronic Inc., and any groups or companies that work with the sponsor, such as PPD Medical Device contract research organization.
- government agencies, such as the US Food and Drug Administration and agencies like it in other countries, or agencies of the Department of Health and Human Services, and
- Scripps committees that review research to help protect people who join research studies.

Once we have shared your information we cannot be sure that it will stay private. If ***you*** share your information with people outside the research team, it will no longer be private. Your name will not be used in any report that is written.

How long will Scripps and Medtronic use and share my information?

- Your information will be used and shared until the research is completed, which we think will be by the end of 2009.

What if I change my mind about sharing my research information?
If you decide not to share your information anymore:

- The sponsor and the research team can continue to use any of the private information that they already have.

- You will no longer be a part of the research study.
- You will still get the same medical care that you've always had at Scripps.
- You must write to the investigator and tell him that you no longer want to share your information. Write to the investigator at:

John Rogers, M.D.
10666 N. Torrey Pines Road SW 206
La Jolla, California, 92037

Do I have the right to see and copy my research information?
You cannot see your research information while the study is going on, unless it is also being used for your health care. Once the study is over, you can ask to see any research information that is in your Medical Record that is kept at Scripps.

If you agree to share your information you should sign this form below. You will be given a copy of this form.

****

I agree to share my information as described in this form

____
Print your name

________________________________ _______________________
Sign your name Date

If you have questions or concerns about your privacy and the use of your personal medical information, contact the investigator at the telephone number listed in the consent form.

VOID
